# The meiotic transcriptome architecture of plants

**DOI:** 10.3389/fpls.2014.00220

**Published:** 2014-06-05

**Authors:** Stefanie Dukowic-Schulze, Changbin Chen

**Affiliations:** Department of Horticultural Science, University of MinnesotaSt. Paul, MN, USA

**Keywords:** meiosis, transcriptome, meiocytes, anthers, RNA-seq, microarray

## Abstract

Although a number of genes that play key roles during the meiotic process have been characterized in great detail, the whole process of meiosis is still not completely unraveled. To gain insight into the bigger picture, large-scale approaches like RNA-seq and microarray can help to elucidate the transcriptome landscape during plant meiosis, discover co-regulated genes, enriched processes, and highly expressed known and unknown genes which might be important for meiosis. These high-throughput studies are gaining more and more popularity, but their beginnings in plant systems reach back as far as the 1960's. Frequently, whole anthers or post-meiotic pollen were investigated, while less data is available on isolated cells during meiosis, and only few studies addressed the transcriptome of female meiosis. For this review, we compiled meiotic transcriptome studies covering different plant species, and summarized and compared their key findings. Besides pointing to consistent as well as unique discoveries, we finally draw conclusions what can be learned from these studies so far and what should be addressed next.

## Introduction

Knowledge regarding meiosis in plants and other species has been obtained over decades (by now even more than a century), mainly from cytological observations and mutant analysis. Many excellent reviews have been written about what is happening in plant meiosis at the chromosome level, and which key players orchestrate recombination, progression and division (for example see Jones and Franklin, 2008; Harrison et al., 2010). However, there is still a lot to explore, especially at the molecular level, facilitated by more recent high-throughput technologies like microarray or RNA sequencing. Massive data obtained by large-scale transcriptome studies allows in depth examinations of enriched processes and of genes that are up-regulated during meiosis, many of which remain uncharacterized.

To gain insight into the gene expression landscape during meiosis, large-scale meiotic transcriptome studies have been performed in various organisms like *Saccharomyces cerevisiae* (Chu et al., 1998; Primig et al., 2000), *Schizosaccharomyces pombe* (Mata et al., 2002), *Drosophila melanogaster* (Andrews et al., 2000), *Caenorhabditis elegans* (Reinke et al., 2000), *Mus musculus* (Pang et al., 2006), and *Rattus norvegicus* (Schlecht et al., 2004). Except in yeast systems, obtaining pure cells undergoing meiosis (meiocytes) can be tricky, and thus, often whole reproductive organs are used. As a consequence, although many transcriptome studies have been performed on reproductive tissues, only a handful used isolated meiocytes. To study gametophyte development, examining transcriptomes of whole reproductive organs is not ideal. An improvement are so-called ablation studies, which profile mutants defective in meiosis in comparison to wild type. This helped to detect key genes for meiosis due to their absent or down-regulated expression in mutants, (Johnston et al., 2007; Ma et al., 2007, 2012; Wijeratne et al., 2007; Nan et al., 2011; Feng et al., 2012; Wei et al., 2013). However, many of these plant studies paid their attention especially to post-meiotic male gametophyte development, starting after meiosis is completed (reviewed in Mascarenhas, 1990; McCormick, 2004; Twell et al., 2006; Becker and Feijó, 2007; Singh and Bhalla, 2007; Borg et al., 2009; Berger and Twell, 2011).

Very little is known of regulatory mechanisms of gene expression during meiosis although a few meiotic transcription factors such as Male Meiocyte Death 1 (MMD1) in *Arabidopsis* (Yang et al., 2003) and Meiosis-associated Zinc-finger Protein (MEZ1) in *Petunia* (Kapoor and Takatsuji, 2006) have already been characterized. Transcriptome profiles of these could provide insight into their target genes but have not been reported yet. In this review paper, we concentrate on the insight we gain from the existent studies that focused on the plant meiotic transcriptome, starting with a brief historical overview.

## Reproductive transcriptome studies

### Historical progress in transcriptome studies

Although we will later focus on gene expression, the term transcriptome extends beyond this and includes different types of RNA that are transcribed; besides the protein coding messenger RNA (mRNA), total RNA contains structural ribosomal RNA (rRNA), transfer RNA (tRNA), and diverse kinds of non-coding regulatory small or microRNA (sRNA, miRNA) as well as long non-coding RNA. Early work on the plant reproductive transcriptome looked at the total RNA amount in developing pollen, using methods like micro-spectrophotometry, autoradiography, and differential extraction of DNA and RNA with perchloric acid at ambient temperatures (Ogur et al., 1951; Taylor, 1953; Woodard, 1958; reviewed in Mascarenhas, 1971). The total amount of RNA per pollen varied between different species, but did so in a reasonable range, with 196 pg in *Tradescantia* (5.1 pg with polyA, equalling ~6 million mRNA molecules; Mascarenhas and Mermelstein, 1981), 230 pg in *Nicotiana tabaccum* (6.2 pg with polyA; Tupy, 1982), and 352–705 pg in *Zea mays* (8.9–17.8 pg with polyA; Mascarenhas et al., 1984). An early study aimed to distinguish between RNA classes occurring in pollen was conducted by Steffensen (1966) in lily: most RNA was found to be synthesized between post-meiotic DNA replication and the first pollen mitosis, with a second smaller peak after pollen mitosis I, and ~75% of the RNA being ribosomal RNA. Thus, studies that measured the whole RNA amount in reproductive tissues reported substantially the changes in rRNA synthesis. To examine gene expression, other studies applied hybridization of 3H-labeled cDNA with RNA in excess (Galau et al., 1976). In contrast to routine large-scale transcriptome analyses done today, this technique was most often performed on polyA-RNA captured from ribosomes, thus giving an even better approximation of which genes are translated and their quantity. Estimated amounts of expressed pollen genes from these experiments (see Mascarenhas, 1975, 1990, 1993) are similar to current numbers obtained with microarray technology (Becker et al., 2003; Honys and Twell, 2003). For maize, Willing and Mascarenhas (1984) reported a total of ~24,000 genes expressed in pollen: 35% of those were very abundantly expressed, including ~240 genes with ~32,000 copies per cell, ~50% of the genes were in the moderately abundant class (~6000 genes with ~1700 copies), and 15% of the genes were in the weakly expressed class (~17,000 genes with ~200 copies); in shoots ~31,000 genes were detected in total, but genes were less abundantly expressed (Willing and Mascarenhas, 1984). For *Tradescantia*, gene numbers reported by Willing et al. (1988) were in a similar range, with ~20,000 genes in total, and again, compared with shoots (~30,000 genes total), the abundant and moderately abundant classes were far larger in pollen (75% of the genes instead of just 35% in shoots). In addition, the average copy number of the low abundant class was far higher (100 copies per cell instead of just 5–10 in shoots). Another lab (Kamalay and Goldberg, 1980), found ~24,000–27,000 transcripts in anther, ovary, leaf, stem, root and petal tissue, and the highest number of specific transcripts (~10,000) in the cases of anther and ovary. They reported that non-translated mRNA is also present in the nucleus, concluding that post-transcriptional regulation might play an important role for gene expression. Taken together, at that time 10–20% of transcripts detected in pollen were suggested to be pollen-specific (Willing and Mascarenhas, 1984; Stinson et al., 1987; Willing et al., 1988).

After these pioneering studies of quantitative assessment of pollen gene expression, studies followed up to identify abundant or specific transcripts by differential screening, sequencing and characterization of cDNA library clones (Koltunow et al., 1990; Scott et al., 1991; Tsuchiya et al., 1994). This method is of course not as high-throughput as RNA-seq or microarray, and usually yielded less than 50 specific cDNAs, but was still used some 10 years ago, e.g., for maize and tobacco generative cell cDNA libraries (Xu et al., 2002; Engel et al., 2003). The real large-scale, high-throughput era only arrived with the emergence of commercial microarrays and RNA-sequencing platforms.

### General remarks—data validity, comparability and usability

**Figure 1:**
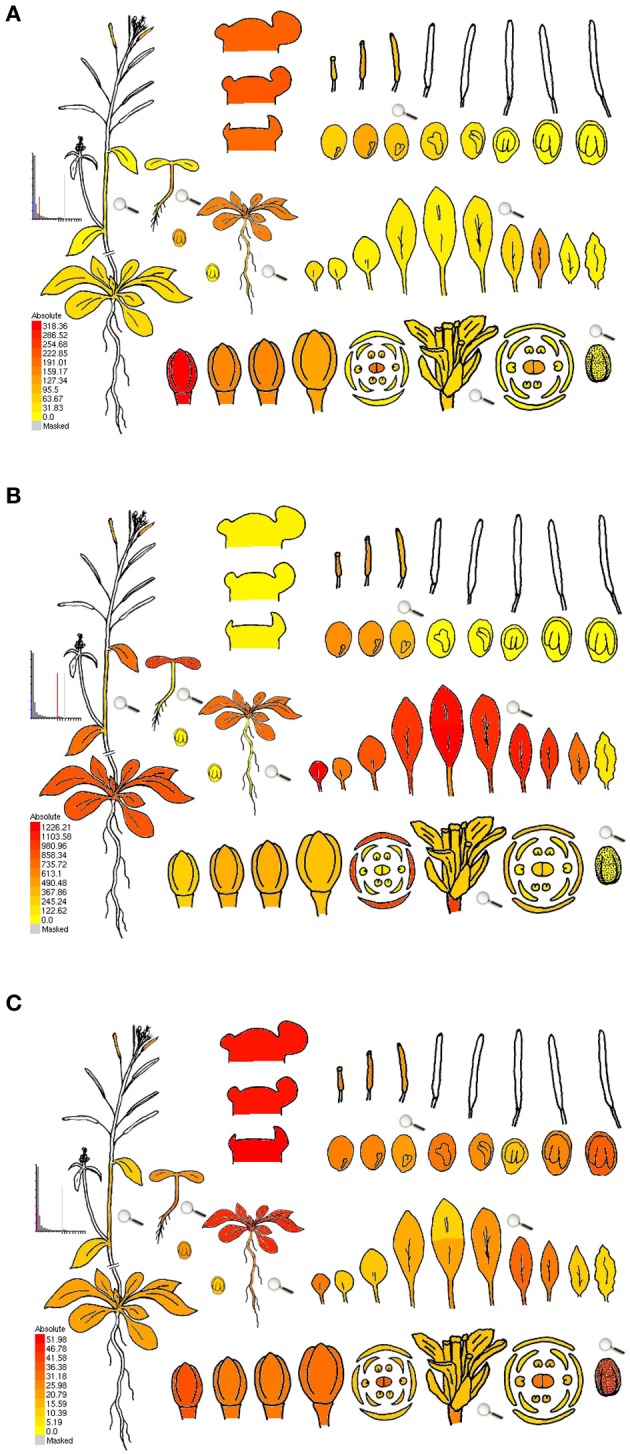
**eFP browser view of gene expression during *Arabidopsis* development. (A)** *DMC1* (At3g22880), encoding a well-characterized meiotic recombinase, confirmed as up-regulated in isolated meiocytes vs. seedlings in *Arabidopsis* and in maize (>5-fold and >1000-fold respectively). **(B)** *NDF1* (At1g15980), encoding a chloroplast NAD(P)H dehydrogenase subunit, identified as more than 35-fold up-regulated in seedlings vs. isolated meiocytes in *Arabidopsis* (and by more than 700-fold in maize homologs). **(C)** *RAD51* (At2g20850), encoding a well-characterized recombinase, operating in both meiotic and somatic tissue; slightly up-regulated in isolated meiocytes vs. seedlings in *Arabidopsis* and in maize (>2-fold and <2-fold respectively). Expression strength coded by color: yellow = low, red = high. The *Arabidopsis* eFP Browser is located at bar.utoronto.ca, published in Winter et al. (2007), developed by B. Vinegar, drawn by J. Alls and N. Provart. Data from Gene Expression Map of *Arabidopsis* Development by Schmid et al. (2005) and the Nambara lab for seed stages.

Advancement of new technologies propelled transcriptome research forward, with Affymetrix developing a microarray gene chip available for the model plant *Arabidopsis thaliana*, covering ~8000 probe sets, followed by the ATH1 gene chip with ~24,000 probe sets (compared in Hennig et al., 2003). Although microarrays allow for comparison between multiple samples, it has to be considered that not all genes might be represented on a chip, and that expression levels between genes are not directly comparable due to different hybridization strengths and background signals (Naef and Magnasco, 2003; Zhang et al., 2003; Gautier et al., 2004). Data analysis involving *t*-tests applied to anthers of different stages from wheat and rice microarray identified ~500 genes putatively involved in meiosis for wheat, in contrast to ~7500 for rice, including only 2 out of ~50 known meiotic genes (Crismani et al., 2011). The authors suggested that this might be due to (i) the wheat genome not being sequenced yet at that time, (ii) specific genes missing on the wheat chip, and/or (iii) to different research groups doing the sampling and processing. In a similar review as our current one, the drawbacks of most studies were mentioned to be as follows: (i) less genes than thought were detected because the ATH1 gene-chip contains mostly sporophytic genes (which can be avoided by using TILLING microarrays instead), (ii) most often whole anthers were used, which might drown gametophyte-specific genes, and (iii) in ablation studies (mutant-wild type comparisons), mutations could cause un-anticipated effects on the transcriptome (Schmidt et al., 2012). The last two drawbacks also apply to RNA sequencing. Hoewver, in contrast to microarrays, RNA sequencing does not rely on hybridization, but is a direct measurement of RNA levels, has excellent technical reproducibility (Marioni et al., 2008; Mortazavi et al., 2008; Bullard et al., 2010), and RNA-seq identifies ~30% more differentially expressed genes in human samples than a standard array (Marioni et al., 2008). Still, there are also minor issues with RNA sequencing, like read number bias due to GC content or fragments with preferential expression (Pickrell et al., 2010; Hansen et al., 2012), making downstream analysis including normalization an important factor. Furthermore, the percentage of alignment to the reference genome depends on RNA and library quality, as well as sequence completeness (Chen et al., 2010). Taken together, results from both microarrays and RNA-seq always depend not only on the quality of the reference genome and gene annotation, but also on the algorithms, software and parameters used, not to mention differences between sampling and processing in different research groups. As an additional note of precaution, we want to point out that neither do transcriptional induction and the amount of transcripts always correlate with protein amount and activity, nor does transcript abundance necessarily imply an essential role of the gene (Kaback and Feldberg, 1985). Nonetheless, transcriptome studies are a valuable tool to detect novel specific or abundant transcripts or prevalent pathways when comparing specific cell types and developmental stages, but have to be connectable with other datasets. Excellent ways to provide this connectivity are publically available datasets of raw data together with detailed description of how they were obtained, as well as studies that produce a whole gene expression atlas, e.g., in rice with 25 reproductive stages (Fujita et al., 2010), and later with 33 laser-assisted micro-dissected anther samples and 143 spatiotemporal samples (Aya et al., 2011), in barley with 15 tissues of different developmental stages (Druka et al., 2006), in *Arabidopsis* with 79 samples through development (Schmid et al., 2005), or in maize with 60 diverse tissues (Sekhon et al., 2011). Data from *Arabidopsis*, maize and more plant species can be easily accessed and mined using the eFP Browser (http://bar.utoronto.ca/welcome.htm; Winter et al., 2007). Figure 1 shows examples of how gene expression details can be examined using the eFP browser, i.e., genes identified in a comparative transcriptome study of *Arabidopsis* and maize data (Dukowic-Schulze et al., 2014a) as up-regulated in isolated meiocytes vs. seedlings (Figure 1A) or the other way around (Figure 1B). Figure 1C shows the well-known recombinase gene *RAD51* which acts in both meiotic and somatic tissue.

## What we have learned from reproductive transcriptome studies so far

### Overview

**Figure 2:**
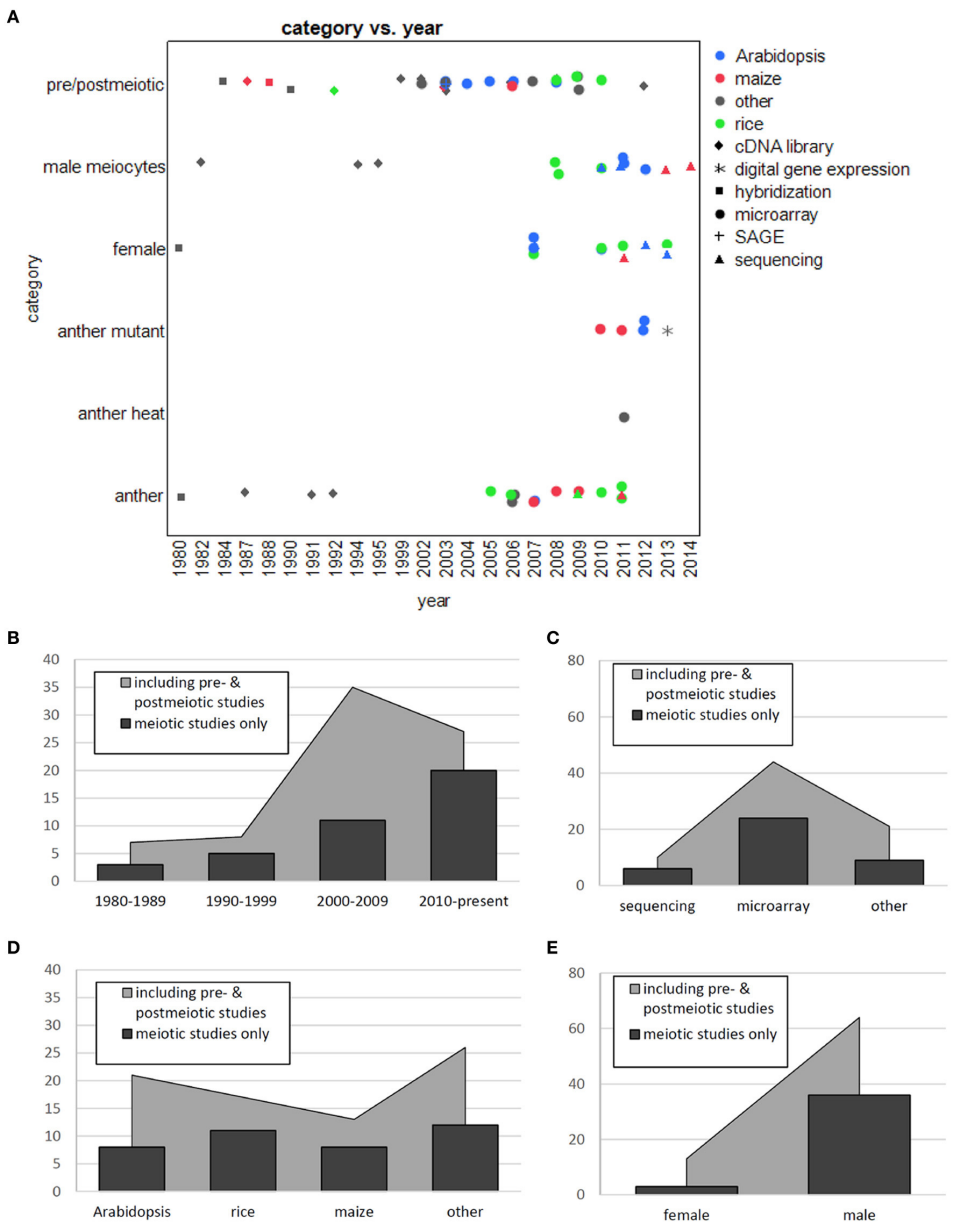
**Distribution of primary literature on transcriptomes of reproductive structures. (A)** Scatter plot with species coded by color, techniques by shape. **(B)** Transcriptome studies per decade. **(C)** Transcriptome studies per technology. **(D)** Transcriptome studies per species. **(E**) Transcriptome studies per gender.

Around a 100 large-scale studies on plant reproductive transcriptomes have been published so far (Figures 2A–E), at least half of them with pre- or post-meiotic samples, many on mature pollen which are easy to obtain (reviewed in Mascarenhas, 1990; McCormick, 2004; Twell et al., 2006; Becker and Feijó, 2007; Singh and Bhalla, 2007; Borg et al., 2009; Berger and Twell, 2011). The remaining around 50 publications focused on or at least included samples during meiosis; 25 studies used whole anthers, 13 studies whole female organs, and 13 studies used isolated male meiocytes (Table 1 and Figure 2). Female studies conducted are almost all on post-meiotic stages, only one study was performed with isolated megaspore mother cells (Schmidt et al., 2011). Whole anthers contain different specialized cell layers surrounding the meiocytes, and the obtained expression profile is thus a mix of these; indeed, in an expression atlas in barley, the anther was found to be the most complex tissue regarding its transcriptome (Druka et al., 2006), and the same was found for maize anthers (Ma et al., 2008). In maize, the examination of different anther developmental stages showed that the transcriptome is most diverse in pre-meiosis (Ma et al., 2007), with reduced expression and few changes during meiosis (Ma et al., 2008). In accordance with this, male *Arabidopsis* meiocytes exhibited less DNA and RNA metabolic activity and more signal transduction when compared to either whole anthers of the same developmental stage or to young seedlings (Chen et al., 2010). Deveshwar et al. (2011) suggested the existence of a two-way molecular switch which shuts down sporophytic genes and activates gametophytic ones toward the end of meiosis. Although most studies were performed on male reproductive transcriptomes, comparison with the few studies from female showed shared and special transcriptome features, as reviewed in Schmidt et al. (2012). Given the excellent reviews on transcriptome studies on post-meiotic gametophytic development (reviewed in Mascarenhas, 1990; McCormick, 2004; Twell et al., 2006; Becker and Feijó, 2007; Singh and Bhalla, 2007; Borg et al., 2009; Berger and Twell, 2011), we concentrate on studies that included or solely used meiotic samples.

**Table 1:** **List of published literature regarding the transcriptome of reproductive stages**.

**Publication**	**Species**	**Technique**	**Sample**	**Cited by**
**MEIOTIC ANTHERS**				
Kamalay and Goldberg, 1980	Tobacco	Hybridization	Anthers	259 (40)
McCormick et al., 1987	Tomato	cDNA library	Anthers	27 (N/A)
Nacken et al., 1991	Snapdragon	cDNA library	Mutant anthers	67 (9)
Scott et al., 1991	*Brassica*	cDNA library	Anthers	112 (14)
Shen and Hsu, 1992	*Brassica*	cDNA library	Anthers	49 (7)
Wang et al., 2005	Rice	Microarray	Anthers	56 (7)
Cnudde et al., 2006	*Petunia*	Microarray	Anthers	12 (5)
Crismani et al., 2006	Wheat	Microarray	Anthers	50 (15)
Druka et al., 2006	Barley	Microarray	Anthers	98 (35)
Lu et al., 2006	Rice	Microarray	Anthers	16 (5)
Ma et al., 2007	Maize	Microarray	Anthers	31 (14)
Wijeratne et al., 2007	*Arabidopsis*	Microarray	Anthers	58 (21)
Ma et al., 2008	Maize	Microarray	Anthers	41 (17)
Huang et al., 2009	Rice	Sequencing	Anthers	40 (9)
Skibbe et al., 2009	Maize	Microarray	Anthers	21 (10)
Fujita et al., 2010	Rice	Microarray	Anthers	41 (14)
Wang et al., 2010	Maize	Microarray	Mutant anthers	12 (7)
Aya et al., 2011	Rice	Microarray	Anthers	8 (3)
Bita et al., 2011	Tomato	Microarray	Anthers heat	13 (5)
Davidson et al., 2011	Maize	Sequencing	Anthers	21 (N/A)
Deveshwar et al., 2011	Rice	Microarray	Anthers	19 (9)
Nan et al., 2011	Maize	Microarray	Mutant anthers	4 (1)
Feng et al., 2012	*Arabidopsis*	Microarray	Mutant anthers	4 (1)
Ma et al., 2012	*Arabidopsis*	Microarray	Mutant anthers	6 (2)
Wei et al., 2013	Cotton	Digital gene expression	Mutant anthers	2 (0)
**MALE MEIOCYTES**				
Appels et al., 1982	Lily	cDNA library	Male meiocytes	21 (N/A)
Kobayashi et al., 1994	Lily	cDNA library	Male meiocytes	95 (11)
Crossley et al., 1995	Lily	cDNA library	Male meiocytes	38 (4)
Hobo et al., 2008	Rice	Microarray	Male meiocytes	71 (26)
Suwabe et al., 2008	Rice	Microarray	Male meiocytes	73 (22)
Chen et al., 2010	*Arabidopsis*	Sequencing	Male meiocytes	28 (12)
Tang et al., 2010	Rice	Microarray	Male meiocytes	23 (8)
Libeau et al., 2011	*Arabidopsis*	Microarray	Male meiocytes	6 (3)
Yang et al., 2011	*Arabidopsis*	Sequencing	Male meiocytes	39 (12)
Barra et al., 2012	*Arabidopsis*	Microarray	Male meiocytes	1 (N/A)
Dukowic-Schulze et al., 2014a	*Arabidopsis*, maize	Sequencing	Male meiocytes	N/A (N/A)
Dukowic-Schulze et al., 2014b	Maize	Sequencing	Male meiocytes	N/A (N/A)
Flórez-Zapata et al., 2014	Sunflower	Sequencing	Male meiocytes	N/A (N/A)
**FEMALE STRUCTURES AND MEIOCYTES**				
Kamalay and Goldberg, 1980	Tobacco	Hybridization	Ovary	259 (40)
Druka et al., 2006	Barley	Microarray	Pistils	98 (35)
Johnston et al., 2007	*Arabidopsis*	Microarray	Mutant pistils	74 (23)
Jones-Rhoades et al., 2007	*Arabidopsis*	Sequencing	Mutant embryo sac	112 (36)
Li et al., 2007	Rice	Microarray	Stigma	67 (20)
Steffen et al., 2007	*Arabidopsis*	Microarray	Mutant ovules	104 (28)
Wuest et al., 2010	*Arabidopsis*	Microarray	Embryo sac cells	100 (35)
Fujita et al., 2010	Rice	Microarray	Ovary	41 (14)
Aya et al., 2011	Rice	Microarray	Ovary	8 (3)
Davidson et al., 2011	Maize	Sequencing	Ovary	21 (N/A)
Schmidt et al., 2011	*Arabidopsis*	Microarray	Isolated MMC	17 (6)
Sanchez-Leon et al., 2012	*Arabidopsis*	Sequencing	Mature mutant ovule	8 (3)
Kubo et al., 2013	Rice	Microarray	Ovule	2 (2)
Armenta-Medina et al., 2013	*Arabidopsis*	Sequencing	Mature mutant ovule	N/A (N/A)
**PRE/POST-MEIOTIC MALE STRUCTURES AND CELLS**				
Willing and Mascarenhas, 1984	*Tradescantia*	Hybridization	Pollen	231 (17)
Stinson et al., 1987	Maize, *tradescantia*	cDNA library	Pollen	148 (18)
Willing et al., 1988	Maize	Hybridization	Pollen	172 (N/A)
Koltunow et al., 1990	Tobacco	Hybridization	Late anthers	644 (93)
Tsuchiya et al., 1992	Rice	cDNA library	Late anthers	47 (5)
Xu et al., 1999	Lily	cDNA library	Sperm cell	98 (16)
Endo et al., 2002	*Lotus*	Microarray	Late anthers	56 (12)
Xu et al., 2002	Tobacco	cDNA library	Sperm cell	40 (N/A)
Amagai et al., 2003	*Brassica*	Microarray	Late anthers	47 (N/A)
Becker et al., 2003	*Arabidopsis*	Microarray	Pollen	267 (77)
Chmelnitsky et al., 2003	Tomato	cDNA library	Late anthers	8 (N/A)
Engel et al., 2003	Maize	cDNA library	Sperm cell	129 (29)
Honys and Twell, 2003	*Arabidopsis*	Microarray	Pollen	371 (112)
Lee and Lee, 2003	*Arabidopsis*	SAGE	Pollen	142 (31)
Honys and Twell, 2004	*Arabidopsis*	Microarray	Pollen	349 (125)
Pina et al., 2005	*Arabidopsis*	Microarray	Pollen	300 (98)
Ma et al., 2006	Maize	Microarray	Early anthers	61 (26)
Mandaokar et al., 2006	*Arabidopsis*	Microarray	Late anthers	141 (39)
Okada et al., 2006	Lily	cDNA library	Sperm cell	30 (4)
Golkari et al., 2007	Wheat	Microarray	Late anthers	65 (4)
Borges et al., 2008	*Arabidopsis*	Microarray	Sperm cell	155 (52)
Hirano et al., 2008	Rice	Microarray	Pollen	75 (22)
Singh et al., 2008	*Plumbago*	cDNA library	Sperm cell	20 (1)
Endo et al., 2009	Rice	Microarray	Late anthers	37 (8)
Frank et al., 2009	Tomato	Microarray	Pollen	59 (18)
Haerizadeh et al., 2009	Soybean	Microarray	Pollen	37 (10)
Wei et al., 2010	Rice	Microarray	Pollen	42 (15)
Ma et al., 2012	Cotton	cDNA library	Late anthers	3 (0)

*Amount of citations till December 2013, as recorded by Google Scholar or PubMed Central (latter ones are in brackets). Only primary literature was used, no reviews*.

### Results and insight from whole anther studies

We will first recapitulate on the insight obtained from whole anther studies. There are additional ablation studies on which we do not elaborate here. These usually focused especially on the genes down-regulated in mutants while we concentrate here on the gene expression landscape in wild-type anthers. Studies using microarray contributed most of the knowledge (Figures 2A,C), but the use of other pioneering techniques earlier on had generated comparable results: Kamalay and Goldberg (1980) had used ribosome-bound mRNA for hybridization with DNA to detect ~26,000 genes in tobacco anthers, of which ~10,000 seemed to be anther-specific. In another non-model organism without available sequence data at that time, Cnudde et al. (2006) used a cDNA-AFLP (Amplified Fragment Length Polymorphism) strategy on meiotic anthers from *Petunia*, and found ~8000 sequence tags of which ~6% were modulated during meiosis, including known meiotic genes.

The amount of genes expressed in anthers was reported as more than 30,000 in maize and wheat (Crismani et al., 2006; Ma et al., 2008; Wang et al., 2010), and ~22,000 in rice (Deveshwar et al., 2011). These numbers vary depending on the stages used for comparison and the criteria used to define expression. More important and informative are lists of genes that are specific to anthers or temporally up-regulated in anthers during meiosis. Of these, 1350 were reported in wheat, 30 of which displayed a more than 8-fold expression change during meiosis, others showing more subtle changes in their expression modulation as seen by hierarchical clustering (Crismani et al., 2006). These weak differences in expression were suggested to be due to the anther dilution effect, and possible additional somatic functions (Crismani et al., 2006). In rice, a similar number, 1000 genes, were found to be anther-specific, most of them due to specificity in mature pollen stages, with only 78 specific during meiosis (Deveshwar et al., 2011). In maize, ~10,000 genes in total might be specific to one or few anther stages, for example ~200 stage-specific genes in 2 mm long anthers (containing cells in meiosis), ~700–900 transcripts shared with the following stage (Ma et al., 2008).

Of the 1350 temporally regulated genes in wheat, many were annotated for chromatin association, synaptonemal complex, recombination, mismatch repair and fertility; and concurrent with anther developmental progression, most functional categories decreased in their expression level, especially meiosis/cell division, ribosomes, transcription factors, organelle activity, signal transduction and lipid and protein metabolism (Crismani et al., 2006). GO (Gene Ontology) terms enriched in genes abundantly expressed in rice anthers during meiosis were transcription factors, protein folding, sorting and degradation, as well as cell structure components (Deveshwar et al., 2011). In maize, transcription factors were prevalent in anthers, and 131 cell wall associated transcripts were found to be enriched (Ma et al., 2008).

Since one intention of these studies was to find out more about meiotic genes, it is interesting which known meiotic genes could be identified by their expression profile. In wheat, the 1350 anther-regulated transcripts included *ASY1, MSH2, 6* and *7, RAD51B* and *C, DMC1*, and *RPA* (Crismani et al., 2006). In rice, *MSH2, 6* and *7, RAD51B* and *DMC1*, as well as *SPO11, MND1, RAD50, ZYP1A/B*, and *MUS81* showed elevated expression levels in meiotic anthers (Deveshwar et al., 2011). In maize, anther genes included genes similar to *EXS1, KU70*, and *MMD1* (Ma et al., 2008), and clustering of genes revealed genes required for meiosis initiation with *DMC1, RAD51* and an *ARGONAUTE/PIWI* homolog (Ma et al., 2007). Interestingly, Deveshwar et al. (2011) pointed out that many meiotic genes such as *HOP2* and *RAD51* were not specifically expressed in meiosis in rice, and concluded that they might play other roles in addition. Another rice study by Tang et al. (2010) on isolated pollen mother cells (PMCs) had found 1158 PMC preferential genes, including many known meiotic genes, but the study on whole rice anthers claimed only 372 core meiotic anther genes since most of the 1158 were also expressed at earlier or later anther stages (Deveshwar et al., 2011).

A data analysis study of the rice and wheat anther data described above found only two out of around 50 known meiotic genes as enriched in both sets concomitantly, namely *ASY1* and *MLH3* (Crismani et al., 2011). Whether other known meiotic genes were missed due to more static expression levels or because they were not included on the wheat chip, a take-home message here is the conserved up-regulation of *ASY1* and *MLH3*, meiotic factors shown to be involved in meiotic recombination and progression (Caryl et al., 2000; Jackson et al., 2006; Sanchez-Moran et al., 2008). Inter-specific conservation of anther-expressed genes could be shown for homologs of maize transcripts in rice and *Arabidopsis* flowers (Ma et al., 2007, 2008), and for some meiotic anther-specific wheat-rice-homolog pairs in reproductive organs of *Arabidopsis* and poplar, though there were exceptions (Crismani et al., 2011). A summary of the efforts to identify early meiotic genes in cereals and an overview table can be found in Bovill et al. (2009). Known genes in early anther development in *Arabidopsis* and maize are also summarized in a supplemental table in Wang et al. (2010). Gene expression during anther development is orchestrated by diverse transcription factors, extensively recounted in a recent review by Khurana et al. (2012). We already mentioned the broad-scale gene expression atlas studies which can be mined for co-expression networks or for detailed information for a gene of interest; in addition, diverse reproductive transcriptomes were analyzed with RNA sequencing in maize (Davidson et al., 2011).

### Results and insight from isolated male meiocytes

In contrast to a vast literature record for mature pollen, there are only few studies that examined the transcriptome of isolated meiocytes. This is probably due to the difficulty and effort involved in obtaining isolated meiotic cells; only recently did feasible techniques become established, such as FACS-based purification techniques applied to pollen (Borges et al., 2012), laser-assisted microdissection (LAM) and capillary collection of meiocytes (CCM) (Schmidt et al., 2012; Chen and Retzel, 2013; Dukowic-Schulze et al., 2014c). An impressive study conducted in lily around 30 years ago is worth mentioning here since it appears to be the first one that used isolated meiocytes for examination of the transcriptome with diverse methods (Appels et al., 1982): autoradiography to detect RNA synthesis of cultured meiocytes implied that most RNA remained on chromosomes in lily pachytene cells, and melting curves of polyA RNA from mitotic and zygotene-pachytene cells were indistinguishable, but little or no ribosomal RNA was detected in meiocytes. Furthermore, they extracted polyA RNA from isolated and cultured meiocytes from interphase till pachytene to generate a cDNA library; of ~5000 cDNA clones, 49 were specifically found in the meiotic samples, and 13 of them were very abundant with 11 sharing a repeated sequence, found to be homologous to sequences of wheat, rye and maize. In addition, they produced polypeptides from lily meiocyte polyA RNA of various stages in an *in vitro* wheat-translation system, which revealed a larger portion of low molecular weight proteins than from somatic polyA RNA samples (Appels et al., 1982). Three more studies on lily meiocytes followed, with Bouchard (1990) continuing analysis of the detected sequence repeats, concluding that these are common with heat-shock proteins. Another lily study used zygotene meiocytes with new cDNA library screening and found 18 genes specific or abundant, including RAD51 related, as well as heat-shock and serine protease genes, many with hydrophobic N-terminus (Kobayashi et al., 1994). Still another lily meiocyte cDNA library was produced from cells ranging from metaphase I to telophase II, and unexpectedly, the 3 clones that were found all expressed far more in tapetal cells than in meiocytes (Crossley et al., 1995). This is a phenomenon also detected in other studies: gene expression from tapetum appears to sometimes (i) contribute overly to the whole anther transcriptome, and also (ii) “leak” to meiocytes, meaning a gene is highly expressed in meiocytes although it has been shown to have a specific function in tapetal cells. Both scenarios can be inspected by *in situ* hybridization for up-regulated genes from either whole anthers or isolated meiocytes (Nacken et al., 1991; Scott et al., 1991; Shen and Hsu, 1992; Rubinelli et al., 1998; Wang et al., 2005; Cnudde et al., 2006; Dukowic-Schulze et al., 2014b). That none of the other lily studies seemed to capture these tapetum-prevalent genes might be dependent on the stages examined: the transcriptome landscape of meiocytes is generally more unique and richer in early prophase stages than in later stages, when the transcriptome activity and complexity in meiocytes decreases (Mackenzie et al., 1967; Crismani et al., 2006; Ma et al., 2008). After these initial studies, the next set of studies used rice: 140 anther-specific genes, identified before in anthers containing pollen (Endo et al., 2004), were further classified by microarray of laser-assisted micro-dissected tapetum and microspores from 5 different developmental stages as 71 being gametophyte-specific, 7 tapetum-specific and 62 common to both (Suwabe et al., 2008). A following publication isolated pre-meiotic PMCs with laser-assisted micro-dissection and analyzed their transcriptome with microarray (Tang et al., 2010). Among the 1158 PMC preferential genes were known meiotic genes such as *SPO11* and *DMC1*, and enriched processes included DNA replication and repair, ubiquitin-dependent proteolysis and MADS-box transcription factors.

Very recent *Arabidopsis* studies concentrated on isolated meiocytes during meiosis, and on what the core meiotic transcriptome can tell us. In all three cases, pools of meiocytes from various stages were collected by micro-capillary collection, and the extracted RNA processed by Illumina sequencing (Chen et al., 2010), SOLID sequencing (Yang et al., 2011), or CATMA microarray (Libeau et al., 2011). In spite of using different technologies and downstream analysis tools, the numbers of genes found up-regulated in meiocytes were quite close, with over 1000 genes in Chen et al. (2010), over 800 in Yang et al. (2011), and 1586 or 2155 (with an overlap of 752 genes in both sets) in Libeau et al. (2011), depending on whether leaves or root tips were used for comparison. Two of the groups reported up-regulated transposable elements (TEs) in meiocytes, ~1000 located preferentially in the pericentromeric region in one case (Chen et al., 2010), vs. a generally low TE activity and only ~50 up-regulated TEs, mostly concurrent with high neighboring gene activity, in the other case (Yang et al., 2011). Such discrepancies between results in different publications can occur, or only one group investigated a specific aspect which was neglected by other groups. Attentive readers might thus have some open questions, and can in case of further interest consult the original data which is usually publically available. Examples for meiocyte transcriptome specialties only reported by one group are the high up-regulation of 55 genes that mapped to a mitochondrion-derived insert on chromosome 2 (Chen et al., 2010), the transcription of introns of more than 400 annotated genes which might be due to annotation errors or special functions (Yang et al., 2011), and the detection of small RNAs (Yang et al., 2011).

One area covered by all three *Arabidopsis* meiocyte studies was the analysis for known meiosis genes. Libeau et al. (2011) inferred that the expression of known meiotic genes is not strongly up-regulated, but that it might be due to a dilution effect from using a population of various stages. An attempt to cluster meiosis genes together was only partly successful, with one cluster (out of 220) with 110 genes that contained 8 meiosis genes. Using a different approach, Chen et al. (2010) found most of the 68 known meiotic genes analyzed to be expressed at 2-fold or higher in meiocytes and/or anthers when compared with seedlings, including prominent players like *SPO11, DMC1, RAD51C, XRCC3, MSH4* and *5, MER3/RCK, PTD, MUS81*, and *SDS*. Looking at known meiotic genes from different perspectives, Yang et al. (2011) found *SDS, RCK*, and *MMD1* included in their meiocyte up-regulated gene list, and expression of many mismatch repair gene family members during meiosis. In addition, they used a broader analysis to compare the genes expressed in meiocytes with their homologs' expression in mouse and yeast and concluded that *Arabidopsis* shows more similarity to mouse than to yeast (Yang et al., 2011). Functional analysis for GO terms enriched in genes up-regulated in meiocytes vs. non-meiotic tissues or whole anthers was carried out in both sequencing-based studies, but with different tools, implying less DNA and RNA metabolism, but more signaling activity regarding biological processes, as well as less kinase but more nucleotide binding activity regarding molecular function (Chen et al., 2010), and pointing to prevalence of transcription factors, cell-cycle progression genes, proteolysis, DNA replication, repair and pairing (Yang et al., 2011).

After these initial studies on isolated *Arabidopsis* meiocytes, an additional study used LCM and microarray to compare the transcriptome of isolated meiocytes between wild type and the histone acetylation mutant *Atmcc1* (Barra et al., 2012). Besides detecting candidate *AtMCC1* target genes, they found up-regulation of *ASK1* and *RAD51C* in the mutant, and effects on expression levels of genes for the meiotic or mitotic cell cycle, proteolysis and chromatin (Barra et al., 2012).

**Figure 3:**
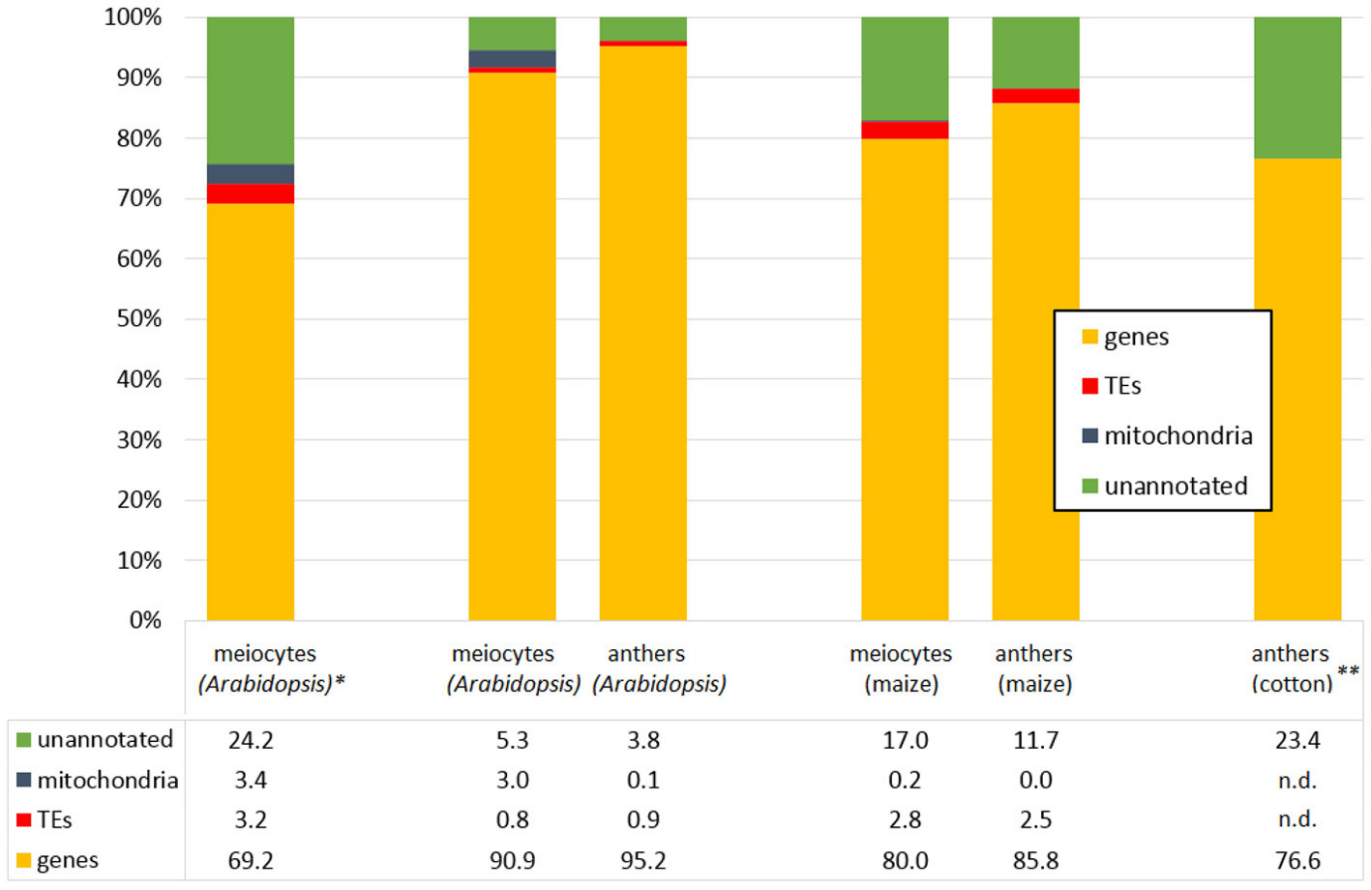
**Example distributions of aligned RNA-seq reads**. Meiocyte or anther RNA-seq reads, mapping to genes, mitochondria sequence, transposable elements (TEs) and unannotated features. Data from Dukowic-Schulze et al. (2014a,b), ^*^Chen et al. (2010), ^**^Wei et al. (2013).

Very recently, comprehensive transcriptome profiling of isolated meiocytes of two other plant species was carried out: in maize meiocytes from early prophase, ~2000 genes were preferentially expressed, most of them uncharacterized (Dukowic-Schulze et al., 2014b). These up-regulated genes included many genes whose products function in mitochondria, and GO analysis indicated enrichment of carbohydrate metabolism, proteolysis, protein targeting, chromatin modification and redox homeostasis. In addition, candidate homologs of most known meiotic genes were up-regulated in meiocytes in comparison to seedlings, for example ~5-fold for *MSH2, RAD51* and *RPA1*, ~10-fold for *ASY1, ZYP1, MND1* and *SPO11*, and even ~1000-fold for *DMC1* (Dukowic-Schulze et al., 2014b). A comparison of this maize data and new *Arabidopsis* data with only early prophase meiocytes from the same lab and methods showed that an increased amount of reads mapped to unannotated features in meiocytes in both species (Figure 3; Dukowic-Schulze et al., 2014a). Furthermore, it could be demonstrated, that mitochondrial transcripts were elevated in both species and derived from the mitochondria themselves. Known meiotic gene homolog-pairs could be clustered together, and common enriched GO terms included organelle organization, protein import, targeting and localization, as well as DNA and double-strand break repair. There were also unique features and GO terms, which might be biologically relevant, but could also be due to differences in annotation quality and quantity. Meiocytes of both *Arabidopsis* and maize were enriched for transcription factors, especially of the MADS-box family, including homeotic genes like APETALA3, PISTILLATA, and AGAMOUS; and in accordance with common transcription factors, promoters of meiocyte up-regulated genes also possessed common *cis*-regulatory motifs (Dukowic-Schulze et al., 2014a), which had also been reported in *Arabidopsis* before (Li et al., 2012).

In contrast to *Arabidopsis* and maize which are excellent model organisms with many resources and a huge research community, another study with isolated meiocytes dedicated its efforts toward a less used and more challenging species, sunflower (Flórez-Zapata et al., 2014). They found 7755 transcripts with higher expression in meiocytes than in somatic tissue. These were enriched for GO terms related to reproduction, and included homologs of genes with known meiotic function. Furthermore, transcription factors specific to meiocytes were detected, as well as many genes related to gene silencing (Flórez-Zapata et al., 2014).

### Results and insight from female pistils and meiocytes

In contrast to the relatively well-studied male meiotic transcriptome, female meiosis was less examined so far, probably due to the difficulties to obtain the corresponding material of whole early pistils or even isolated megaspore mother cells. One study examined the stigma transcriptome (Li et al., 2007), and genetic ablation was used to study genes preferentially expressed in the mature ovule (Sanchez-Leon et al., 2012; Armenta-Medina et al., 2013) and in the mature embryo sac (Johnston et al., 2007; Jones-Rhoades et al., 2007; Steffen et al., 2007). Furthermore, individual cell types in the mature embryo sac were examined (Wuest et al., 2010; Ohnishi et al., 2011), but all these studies looked at post-meiotic transcriptomes.

A study focused on the meiotic transcriptome was performed by applying microarray on laser-dissected megaspore mother cells (MMCs) of *Arabidopsis*, which indicated the expression of ~9000 genes, including known key meiosis genes like *MLH1, MSH4, REC8/SYN1, PTD1*, and *TAM* (Schmidt et al., 2011). Around 800 genes were significantly up-regulated in MMCs, enriched for processes related to structural components and ribosome biogenesis, translation control, ion transport and chromatin constituents. Another, very recent study was performed with whole rice ovules of different stages containing MMCs up to the mature embryo sac, including integument tissue, which still required laser-dissection (Kubo et al., 2013). They found ~37,000 probes expressed in ovules, ~450 only in ovules, including 8 cytochrome P450 genes and 24 transcription factors. In total, 85 transcription factors were up-regulated in ovules, especially of the MADS-box, AP2-EREBP and ABI3VP1 families. Known meiosis-related genes were found, e.g. *DMC1B, MEL1, MSP1, SDS, PAIR1*, and 3 were highly expressed during both male and female meiosis, and *DMC1A, MEL2, MER3/RCK, TOP6A, PAIR2, ZEP1*, and *RAD21-4* showed lower and more constant expression in ovules than in anthers. Since they examined different stages, they could show that a major down-regulation of genes takes place between zygotene-pachytene and later stages, with many genes more than 10-fold decreased, which might be due to genome-wide silencing. During pachytene, a temporal and dramatic up-regulation of a subgroup of TE elements was detected, especially of the mudrA-like, snapdragon TNP2-like and retrotransposon-like classes. After pachytene, genes involved in epigenetic modifications that might be involved in TE repression were up-regulated namely *MET1-2, MET2a*, and *RDR3* (Kubo et al., 2013).

### Special types of RNA

We mentioned in a previous paragraph that the earliest studies on reproductive plant tissue measured whole RNA content, and that most of it was due to rRNA. Interestingly, there appears to be an oscillating production curve for rRNA. An observation that was made by molecular and cytological means during meiosis was that the total RNA content decreases in prophase I till diplotene/diakinesis, and increases again afterwards; in accordance with this, pachytene cytoplasm showed fewer ribosomes than for example pre-leptotene (Mackenzie et al., 1967). The ribosome depletion during meiosis has also been observed by other groups (Williams et al., 1973; Stern and Hotta, 1977; Dickinson, 1987).

Most rRNA synthesis in microspores was detected between post-meiotic DNA replication and pollen mitosis I in lily, with a second but smaller peak after pollen mitosis I (Steffensen, 1966). Another study had also shown an increase in total RNA late in pollen maturation, and that total RNA content was higher during late meiosis than during the first free microspore stages (Ogur et al., 1951). A similar ribosome synthesis pattern was seen in *Tradescantia* with most RNA synthesis before anther dehiscence (Mascarenhas and Bell, 1970). Mascarenhas (1990) confirmed that rRNA transcription happened prior to pollen mitosis I and that no synthesis occurred in mature pollen or the developing tube in both *Tradescantia* and lily. The pattern of tRNA synthesis was similar (Peddada and Mascarenhas, 1975). In agreement with low or no rRNA synthesis in mature pollen, nucleoli—the structure where rRNA transcription takes place—are very small or absent late in pollen maturation in *Tradescantia* (Woodard, 1958). Taken together, the described oscillation of rRNA synthesis and related structures is very intriguing, and might imply changing requirements and resource allocation during pollen development.

While rRNA production was investigated more thoroughly early on, recent interest shifted to another transcriptome component, namely small RNAs. These small regulatory molecules, connected with silencing or gene regulation in general, were shown to affect both male and female reproduction (Millar and Gubler, 2005; Wu et al., 2006). Some of the transcriptome studies on female meiosis examined small RNAs during megasporogenesis (Schmidt et al., 2011), and found enrichment for PAZ and PIWI domain proteins in egg cells (Wuest et al., 2010). Studies on post-meiotic male gametophyte development detected the presence of small RNAs and proteins involved in small RNA pathways (Chambers and Shuai, 2009; Grant-Downton et al., 2009a,b; Slotkin et al., 2009; Wei et al., 2010). These and other studies already revealed exciting possibilities for regulation of post-meiotic gene expression and transposable element silencing, and the interested reader is referred to comprehensive reviews on this topic (Borges et al., 2011; Trionnaire et al., 2011; Van Ex et al., 2011; Gutierrez-Marcos and Dickinson, 2012; Feng et al., 2013). Here, we concentrate our attention on findings from studies during meiosis, though only few clues are existent at this time, most of which do not stem from large-scale transcriptomic analyses: AGO1 is one of the main regulators of microRNA (miRNA, 21–24 nucleotides long) directed target cleavage in *Arabidopsis*, and mutants show multiple developmental defects, including sterile flowers in some instances (Vaucheret et al., 2004). An *AGO5* homolog in rice, *MEL1* was shown to be required for correct progression through meiosis (Nonomura et al., 2007). *AGO9* is required to specify cell fate in *Arabidopsis* ovules, controlling the amount of gametes (Olmedo-Monfil et al., 2010), and a mutant of an *AGO9* homolog in maize could produce gametes without meiosis, while showing chromatin condensation defects and failure in chromosome segregation in meiosis (Singh et al., 2011). Taken together, the progress in research of small RNA pathways resembles the one for meiotic genes: at first, mutant analysis supported the characterization of single genes, now moving to large-scale approaches. A few recent studies on meiotic transcriptomes mentioned small RNAs or *ARGONAUTE* genes: in whole anther transcriptome studies, Ma et al. (2006; pre-meiotic), as well as Huang et al. (2009; including meiotic anthers) detected antisense transcripts at around 10% of sense transcripts level, suggesting their role to be regulatory or RNA splice intermediates. One of the studies conducted on isolated male meiocytes in *Arabidopsis* (Yang et al., 2011) reported miRNAs, for example mi163, which was shown to act against SAM-dependent methyltransferases (Allen et al., 2004); it was pointed out that the approach can only detect small RNAs with polyA, so called pri-mRNA (Kurihara and Watanabe, 2004). In the transcriptome of isolated male meiocytes, another clue for the importance of small RNA pathways was found, since *AGO3* and *AGO8* were highly expressed in meiocytes (Chen et al., 2010).

## Beyond the transcriptome—what's next?

When writing this review, we observed a trend that studies done around the same time focused on a certain area. In the very beginning, most attention was on rRNA, which is now not thoroughly examined in the context of meiosis anymore. Then, different techniques enabled mRNA-focused studies. While the first of these were done on meiocytes or reproductive organs during meiosis, afterwards for around a decade around the turn of the century, there was a substantial gap before attention shifted back to meiosis (Figures 2A,B). In the meantime, lots of transcriptome studies had focused on pre- or even more frequently on post-meiotic reproductive organs and processes.

With this in mind, we want to share our thoughts about where the future of meiotic studies will probably lead us. One big emerging topic surely are regulatory RNAs, encompassing both long non-coding RNA and small RNA. Many different kinds of small RNAs exist, defined in part by their nucleotide length, and they act in multiple roles in gene regulation. Sequencing of small RNA from meiotic stages will certainly give us even more data that can be integrated into whole regulatory networks. As a proof of concept, pioneer studies on small RNA profiling in plant reproductive tissue have been already performed on mature pollen and mature ovules, revealing novel regulation mechanisms in these late stages (Slotkin et al., 2009; Olmedo-Monfil et al., 2010). Further approaches which can help elucidate the underlying mechanisms of the meiosis transcriptome architecture include profiling of epigenetic marks such as DNA methylation and histone modification, and should be performed with isolated meiocytes in the future.

Although a lot of reproductive transcriptome data have been produced till date, the data from large-scale transcriptome studies are neither fully exploited nor should they be seen as the gold standard for high-throughput examination of gene expression: first, conclusions that can be drawn reproducibly from different species should be pursuit in more detail, like the enrichment for certain transcription factors and *cis*-regulatory elements, proteolysis as an important regulatory component, and the abundance of mitochondrial transcripts. Discoveries made in one or multiple species should be verified by re-analyzing more data sets simultaneously, and then examining more details by exploiting the data for this particular aspect and adding additional experiments such as those involved in mutant characterization. Second, we should keep in mind that we cannot absolutely rely on conclusions we draw from mere polyA RNA-sequencing data because (i) the splicing landscape might be globally reprogrammed as in mouse meiosis (Schmid et al., 2013), having consequences for expression or function, and (ii) translation regulation can still change the resulting amount of gene products, as an translational initiation factor in *Drosophila* spermatogenesis was shown to be important for segregation, cytokinesis and shaping protein levels (Hernández et al., 2012). Two approaches to get closer to the relevant gene expression are ribosome profiling and proteomics—both challenging due to the difficulty to obtain enough pure meiotic material. An extensive ribosome profiling study in yeast investigated RNA bound to ribosomes at 25 time points during meiosis (Brar et al., 2012), and for ~200 genes they demonstrated an ~10-fold change in translation efficiency (calculated by dividing the reads per kilobase per million mapped reads—RPKM—from ribosome-bound RNA by the RPKM of total polyA RNA). Besides the very early studies done in lily and tobacco, to our knowledge no newer large-scale plant meiotic study has used ribosome-profiling. The second approach to get more insight into processes occurring during meiosis is to look even further, to the protein level. Indeed, first proteomics studies have been conducted in meiotic anthers and pollen, and though their resolution is not that high yet, they yield valuable information about major factors present during meiosis. As in the case of transcriptome studies, most proteomics studies used mature pollen (Holmes-Davis et al., 2005; Noir et al., 2005; Dai et al., 2006, 2007; Sheoran et al., 2006, 2007; Lopez-Casado et al., 2012). However, two associated studies in rice included early anthers containing meiotic cells (Imin et al., 2001; Kerim et al., 2003), and found global differences in the proteome during microspore development, with 150 proteins identified. An interesting aspect was revealed by Wang et al. (2010), who performed both transcriptomics and proteomics on anthers and found additional changes in timing or differential expression when looking at the proteome instead of the transcriptome.

## Concluding remarks

Why are all these transcriptome studies of reproductive plant tissue important? Besides the academic questions about how organs as sophisticated as anthers and pistils arise, develop, function, and are regulated, there are also direct uses feasible for horticulture and agriculture, namely breeding and ensuring crop production. Both are intertwined—basics have to be understood and unraveled by fundamental research, and real-life observations, issues and goals can lead to more application-based research, aiming toward better selection and manipulation of plants.

Examining the effects of extreme temperatures on meiosis is one example and appealing for two reasons, (i) climate change (especially since elevated temperature can cause sterility and thus result in crop yield reduction), and (ii) recombination-pattern changes in elevated temperatures (Lu, 1974; Higgins et al., 2012), which could lead to broader variety in plant phenotypes for breeding. A recent review (Giorno et al., 2013) nicely sums up the findings from transcriptome studies of plant reproductive tissues where heat was applied. The amount of genes that was found to be differentially regulated by temperature influence in the anther ranged from 31 to 3353, and might contain candidates for targeted breeding to cope with changing climate.

Facilitating hybrid creation with a male-sterile parent can be accomplished by genetic engineering for male sterility. For this, knowledge is needed about candidate genes essential for male fertility as well as meiosis-specific promoters. Some studies already followed up on large-scale transcriptome profiling to identify and test meiosis-specific promoters (Steffen et al., 2007; Li et al., 2012).

Although there are a lot of published studies on the plant meiotic transcriptome by now, most of the obtained data are not yet exploited to a satisfactory level. Besides the promoter studies mentioned above, another study followed up on high expression of *AGO9* in *Arabidopsis* ovules with a detailed cytological characterization and sequencing of AGO9-associated small RNAs (Olmedo-Monfil et al., 2010). We hope to see more research of this kind in the future, that examines existent data sets and focuses on specific findings to reveal more detail. In addition, there is a need for more studies that compare conclusions from different studies, concentrating on conserved findings as well as discrepancies that might be interesting to pursue by first re-examining raw data.

What is now needed are more specific (meaning single gene or process) examinations, further aspects (like ribosome profiling, small RNA sequencing and proteomics), as well as improved connectivity (between different species and platforms). In addition, far more studies address male meiosis than female meiosis. For the future, research will hopefully catch up on neglected aspects like female meiosis, the differences in gene expression and processes between male and female meiosis, and the importance or consequences of up-regulated transposons or mitochondria genes. We are looking forward to a time of combined efforts that will hopefully lead to better fundamental understanding of meiosis, and to innovative applications and adaptations to ensure meiotic success in a changing environment.

### Conflict of interest statement

The authors declare that the research was conducted in the absence of any commercial or financial relationships that could be construed as a potential conflict of interest.
